# Reply to Bourgeois et al., “Incompletely Reported Important Methodological Details and Inaccurate Description of the Formulation That the Control Arms Received in a Gardasil Vaccine Trial”

**DOI:** 10.1128/mSphere.01010-20

**Published:** 2020-11-04

**Authors:** Suzanne M. Garland, Marc Steben

**Affiliations:** a Department of Obstetrics and Gynaecology, University of Melbourne Medical School, Faculty of Medicine, Dentistry and Health Sciences, Melbourne, Australia; b Canadian Network for HPV Prevention, Montreal, Canada

**Keywords:** placebo aluminum, safety HPV vaccine

## REPLY

Bourgeois et al. (1) write with concerns of what they perceive as “inaccurate and incomplete reporting, as well as ethical concerns about the informed consent process,” of a trial published in 2007 by ourselves (2).

First, we wish to dispel any suggestion that the authors were not transparent about the fact that the placebo used in the study contained aluminum. Second, it was specifically so stated in the article. Third, it was specifically so stated in the informed consent form. We address these points in more detail below under safety, informed consent, and placebo.

## 

### Safety.

At the time of publication, the quadrivalent vaccine, Gardasil, had been on the market for 1 year (i.e., since 2006). The nine-valent vaccine, Gardasil 9, was approved in 2014 and is currently approved in more than 80 countries and regions.

Gardasil and Gardasil 9 are the combined result of nearly 20 years of research and development. Together, the safety and efficacy of these vaccines have been established in 19 phase 3 clinical trials involving more than 49,000 females and males, aged 9 to 45 years old. Safety has continued to be evaluated in several postlicensure surveillance studies in approximately 4.7 million people collectively.

Multiple independent scientific organizations and major regulatory and public health authorities, including the World Health Organization (WHO), the U.S. Centers for Disease Control and Prevention (CDC), the U.S. Food and Drug Administration (FDA), the European Medicines Agency (EMA), and Health Canada, among others, have repeatedly evaluated safety of human papillomavirus (HPV) vaccines over time. Findings from many vaccine safety monitoring systems and more than 160 studies have shown that HPV vaccines have a favorable safety profile—the body of scientific evidence overwhelmingly supports their safety (3).

### Informed consent.

As you will be aware, informed consent is required for participation in FDA-regulated clinical investigations (3). Merck clinical trials and clinical trial investigators adhere to and comply with FDA guidance and requirements regarding informed consent. As such, investigators conducting clinical trials on behalf of Merck do not involve a human being as a subject in research covered by these regulations, unless the investigator has obtained the legally effective informed consent of the subject or the subject's legally authorized representative. As one of us was the principal investigator for FUTURE I in Australia, the trial was approved to proceed only after being reviewed in the Royal Women’s Hospital Research and Ethics Committees. Informed consent ensures that study subjects are made aware of the nature of the study and study protocol reflecting true risk, therapy, and toxicity.

The patient information and consent form (PICF) used by clinical trial investigators for the FUTURE I Study, and that given to and signed by all participating subjects, clearly states that the HPV vaccine placebo would be an inactive solution (containing aluminum 225 μg/dose) and that the placebo contained no active vaccine.

### Placebo.

In clinical trials, the type of control treatment may be any of the following: (i) placebo, (ii) no treatment, (iii) different dose or regimen of the study treatment, or (iv) a different active treatment (4). In a placebo-controlled trial, subjects are randomly assigned to a test treatment or to an identical-appearing treatment (as was the case with FUTURE I) that does not contain the test drug. Such trials are usually double blind. The placebo control design, by allowing blinding and randomization and including a group that receives a treatment without the test drug, controls for all potential influences on the actual or apparent course of the disease other than those arising from the pharmacologic action of the test agent (5).

As mentioned above, the HPV vaccine placebo in the FUTURE I Study was an inactive solution (containing aluminum 225 μg/dose). The placebo contained no active vaccine. Of note, the most common source of exposure to aluminum is from eating food or drinking water, albeit by the oral route (6).

In addition, the FUTURE I study has been described in peer-reviewed publications as a placebo-controlled study, and the placebo was clearly and correctly described as aluminum-containing placebo (see, for instance, references [Bibr B7][Bibr B8][Bibr B9]) in the same way it is described in the publication in *Clinical and Vaccine Immunology*. The 2007 *New England Journal of Medicine* article (7) under “Methods,” p. 1929, clearly states (under “Vaccine and Randomization”): “The quadrivalent HPV-6/11/16/18 L1 virus-like–particle vaccine with amorphous aluminum hydroxyphosphate sulfate (Gardasil, Merck) as an adjuvant and the aluminum-containing placebo were visually indistinguishable and have been described elsewhere.”

We disagree entirely with Bourgeois et al.’s unsupported claim that the rationale for using a placebo with aluminum “lacks clinical relevance.” The paper stated that the “aluminum-containing placebo [has] been described previously” and provided a citation to a paper that explained the use of a placebo with aluminum was “for appropriate safety comparisons” (10). Moreover, aluminum has been utilized in vaccines for over 6 decades.

Thank you for the opportunity to provide more clarity about this study and further confirm that it was conducted safely, effectively, and in compliance with all global regulations.
